# The Effect of Disinfection with Sodium Hypochlorite 0.5% on Dimensional Stability of Condensation Silicone Impression Materials of Speedex and Irasil

**Published:** 2014-09

**Authors:** Mohammad Hasan Kalantari, Afsaneh Malekzadeh, Ameneh Emami

**Affiliations:** a Dept. of Prosthesis, School of Dentistry, Shiraz University of Medical Sciences, Shiraz, Iran.; b Dentist, Private Practice, Tehran, Iran.; c Periodontist

**Keywords:** Sodium hypochlorite, Condensation silicone, Disinfection, Dimensional changes

## Abstract

**Statement of the Problem:** Impression materials are concerned as a significant source of cross-contamination because of exposure to blood and saliva.

**Purpose:** Considering the importance of infection control in the dental environments, this study is performed to investigate the dimensional changes of two condensation silicone impression materials, Speedex and Irasil, after immersion in 0.5% sodium hypochlorite.

**Materials and Method:** In this *in-vitro* study, two condensation silicone impression materials, Speedex and Irasil, were used on a prefabricated metal model having two dies, one with and the other without undercut. Each impression material was used to prepare 30 impressions; half of each group was immersed in 0.5% sodium hypochlorite for 20 min. The casts were prepared and a profile projector was used to measure the casts in terms of height and diameter of the die without undercut, distance between the two dies, die diameter below the undercut, and the height of the die above the undercut. The results were statistically analyzed using Student t-test.

**Results:** In Speedex group, an increase was detected in the height of die without undercut and the height of the die above the undercut, but other dimensions have decreased. No significant change was observed in dimensions of Speedex group except for the distance between the two dies and die height above the undercut. In Irasil group, the height of the die without undercut, the distance between the two dies and the height of the die above the undercut have increased; while decrease was observed in other dimensions. Compared with the original sample, no significant difference was observed in dimensions except for the height of the die above the undercut.

**Conclusion:** These changes for Speedex group include changes in distance between the two dies and the height above the undercut which can impede proper placement of prosthesis, particularly fixed partial dentures in which the accuracy of the distance between the two dies are of utmost importance. In Irasil group, the height of above the undercut was altered and this negative effect is highlighted in registering the reduced single tooth with an undercut.

## Introduction

Impression materials and appliances used in oral environments such as prostheses can be potential sources of infection transmission. The delivery of contaminated items into the laboratory environment transmits the infection to dental prostheses and the equipments used for other patients. It also increases the danger of infection transmission to the personnel of laboratories [1]. Washing dental impressions with water only partly cleanses the flora on dental impressions.

American Dental Association (ADA) recommends the impressions to be washed with water in order to clean them of blood, saliva and food debris; then be disinfected and sent to laboratory [2]. Although disinfection of impressions eliminates the microorganisms off their surface, dimensional changes can also take place due to chemical or physicochemical reactions between the material set and the disinfectant solutions [3].

Since all the steps in the process of fabricating a precise prosthesis must be performed with ample accuracy and as impressioning is among the most important stages of prosthesis fabrication, infection control procedure must be accomplished in such a way that the impression materials do not undergo dimensional changes.

Many researches have focused on dimensional changes of impression materials after disinfection; while only a few researches have been done on condensation silicones which are extensively used in Iran’s dental offices. Considering the potential of spreading infectious diseases by neglected dental procedures, this study that pursues the study of Safari et al. [4]; aims to investigate the dimensional changes of two condensation silicone impression materials; Speedex (Asia Chemi Teb Co; Tabriz, Iran, under the license of Coltene-Switzerland) and Irasil (Golchai, Iran) after disinfection with sodium hypochlorite 0.5% by employing the immersion method.

## Materials and Method

To evaluate the dimensional stability of condensation silicone impression materials (putty-wash type), the desired dimensions of the samples were measured after immersing in sodium hypochlorite 0.5% and were compared with the impressions produced from experimental (lab) model. This in vitro research was performed on prefabricated and quasi-experimental models. Ease of use, high-precision measurements and reduced intervening variables are the advantages of this method which promoted it to be used in several studies [5-7].

**Figure 1:**
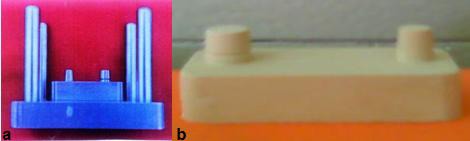
a: Stainless steel model with two dies: with undercut and without it on metal base b: sample of stone cast

The employed model consisted of a stainless steel plate being located on a metallic base made of the same metal, and two dies with convergence of 3° on this base, one without undercut-similar to the prepared premolar tooth (with round cross section)- and the other with undercut (grooved)- similar to the prepared premolar teeth (with the same cross section) (Figure 1a). Four guiding lines were drawn on the main plate with equal distances around base in order to get a smooth placement path for the tray in all impressions. The employed model also had a spacer made of the same material. The impression trays were rectangular cuboids (8×6×5) made of transparent compact plastic (Acrylic Glass).

Numerous holes were created on the upper and lateral faces of the tray for retention of the impression material, creation of way out and reduction of internal pressures of impression material. Moreover, to obtain better adhesion, all impression trays were covered with a thin layer of adhesive material. Impression materials used in this study included Speedex and Irasil condensation silicones which are enormously used in Iran’s dental offices because of their low price, availability and ease of access. The engaged impression technique was space-including technique; the proper and necessary space for the wash material was 1.5 mm, which was provided by a metal spacer, as previously described. Since there is enough space for wash material and it protects the putty material against the stress exerted, the least dimensional changes are present in this technique.

A wide range of disinfectants are used to disinfect the dental impressions and the most common of them is sodium hypochlorite [8-14]. With regards to the established scientific characteristics, economical availability and frequent clinical usage of this material, sodium hypochlorite 0.05% was used as disinfectant material in this study (household bleaching solution diluted 10:1).

Disinfection of impressions can be done with two methods: immersing or spraying. It is claimed that immersion, in recommended duration, would not lead to destructive dimensional changes [15-16].

During impression, first the spacer was placed on the dies and impression was performed with putty material. When the putty material hardened, the tray was separated off the spacer and the plate. After the spacer was removed, the final impression was performed with wash material. A total number of 30 impressions were made using the same technique. For both types of condensation silicone materials, functional properties of putty and wash were considered according to the instructions of the manufacturer.

After completing the time of hardening, prepared impressions were rinsed with lukewarm water and were kept in laboratory environment for 30 minutes. Then 0.05% sodium hypochlorite solution was prepared and after 30 minutes, the impressions were placed in disinfectant solution for 20 minutes. All impressions were casted with Stone Type IV (Bego) according to the instructions of the manufacturer with proper ratios.

**Figure 2:**
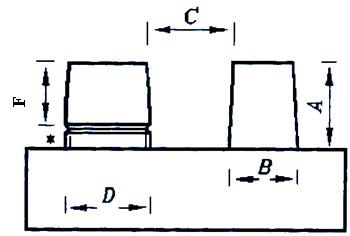
Schematic picture of stone cast showing and illustrating dimensions measured.

The prepared stone was poured into the impression by slow vibration within 3 minutes. After 24 hours, stone casts and the impressions were separated. Finally, 30 stone samples were prepared and numbered randomly (Figure 1b). The following dimensions were measured by a blind specialist using profile projector (Nikon Model6G; Tokyo) with micron- level accuracy (0.001 mm): the height of the die without undercut (A), die diameter without undercut (B); distance between the two dies (C), die diameter below the undercut (D), and die height above the undercut (E) (Figure 2).

## Results

The dimensions of original model and stone casts were measured by Profile Projector device after immersion. Mean, standard deviation and percentage of dimensional changes of the two impression material groups were compared (Table 1 and 2); t-test revealed the following results:

**Table 1:** Means (X̄) and standard deviations and dimensional changes (α) of Speedex

Impression material	Dimension	A	B	C	D	E
Condensation silicone of Speedex	Die model	10.373	7.935	31.332	9.970	6.552
	X̄	10.378	7.913	31.278	9.916	6.605
	SD	0.041	0.0434	0.0437	0.740	0.0433
	α	0.048	-0.277	-0.172	-0.541	0.808

**Table 2:** Means (X̄) and standard deviations and dimensional changes (α) of Irasil

Impression material	Dimension	A	B	C	D	E
Condensation silicone of Irasil	Die model	10.373	7.935	31.332	9.970	6.552
	X̄	10.378	7.913	31.278	9.916	6.605
	SD	0.041	0.0434	0.0437	0.740	0.0433
	α	0.048	-0.277	-0.172	-0.541	0.808

**Height of the die without undercut (A):** In Speedex condensation silicone group, it had shown to have increased before disinfection in similar study, showed decrease after disinfection in the current study, neither of which was statistically significant. In Irasil group, this distance showed to have decreased before disinfection, while an increase was detected in this study; however, none of them was significant. The difference in the height of the die without undercut between two groups of condensation silicones (Speedex and Irasil) that was significant before disinfection, showed no significant difference after immersion (*p*= 0.5).

**Die diameter without undercut (B):** In both groups of condensation siliconee Moreover, the difference between two groups was not significant before and after disinfection (*p*= 0.8).

**Distance between the two dies (C):** In both groups of condensation silicones (Speedex and Irasil) before immersing, a non-significant increase was reported, while in this study a decrease was noticed after disinfection that was statistically significant in Speedex but non-significant in Irasil group. The difference between the two groups that was not significant before disinfection, turned out to be statistically significant after disinfection (*p*= 0.4).

**Die diameter below the undercut (D):** In both groups the diameter of die below the undercut had non-significant changes before immersion, which was detected in form of increase in Speedex and decrease in Irasil. These changes brought about decrease in both groups that unlike the Speedex group, it was statistically significant in Irasil. Although this difference was not significant between the two groups before and after the disinfection (*p*= 0.5).

**The height of die above the undercut (E):** In both groups of Speedex and Irasil, it had non-significant decrease before immersion; but it showed significant increase after disinfection. This difference was not significant between the two groups before and after disinfection (*p*= 0.7).

## Discussion

By evaluating and comparing the dimensions (A, B, C, D, E) of the original sample and Speedex impression material, it was concluded that the die height above the undercut (E) had increased and other dimensions had decreased; and that according to t-test, no significant change was observed in any dimension except in the distance between the two dies and die height above the undercut.

In the group of Irasil condensation silicones, the height of the die without undercut as well as the height of the die above the undercut showed increase; while other dimensions had decreased. The dimensions had no significant difference with original sample excluding the die diameter below the undercut and the height of the die above.

In a study carried out by Sadr and Nasser Khaki on the effect of sodium hypochlorite, a condensation silicone impression material (Optosil-Xantopren) and an addition silicone material (President) were used and their laboratory model was the same as the current study [17].

The results obtained for Optosil-Xantopren showed that the height of the die without undercut and the height of die above the undercut have decreased and the other dimensions have increased. This finding does not conform to the result of the current study, except for the increase in the height of die above the undercut in Speedex group.

The results obtained from Rapid-President imply increase in the height of die without undercut and the height of die above the undercut and decrease in other dimensions. It totally conforms to the results of both groups in our study, excluding the height of the die without undercut in the Speedex group [17].

As mentioned, accurate impressions of prepared teeth result in precision stone casts and proper prosthesis. Any changes in dimensions can lead to improper prosthesis and incomplete seating of crowns and bridges. Significant changes in distance between the two dies that occurred about Speedex condensation silicone material result in distortion and inaccurate registration between adjacent prepared teeth. These changes will be dramatic when impression of adjacent teeth was required for fixed partial prosthesis. These situations lead to improper seating of prosthesis in all abutments or some of them. According to this study, Speedex cannot be suitable for impression making to construct fixed partial prosthesis (bridges) after disinfection by 0.5% sodium hypochlorite, but not about Irasil.

Changes in die height under the undercut can lead to inaccurate crown height (increasing in this study) that occurred in both Speedex and Irasil condensation silicones. When this happens, final restoration will be made in over height that interferes with occlusion and needs more occlusal adjustment, so it has more risk for fracture failure. The width of the die under the undercut showed significant dimensional shrinkage, leading to prosthesis with less width in margin, which results in incomplete sitting of restorations. Consequently, proximal and occlusal contacts of restorations will be disturbed.

In another study that was previously referred to as the basis of this study, Kalantari et al. [4] investigated the dimensional accuracy of condensation silicone impression materials (Speedex and Irasil), using the same model. Conformity was observed in changes of neither group, excluding the diameter of the die below the undercut in Irasil group. In fact, the change of the distance between the two dies in Speedex group was non-significant before disinfection, which became significant after disinfection. It can interfere with the recorded distance between the teeth prepared for fixed partial prostheses.

In Irasil group, die diameter below the undercut that had decreased before and after disinfection, was non-significant at the beginning; but it became significant after immersion. It can be a barrier for the proper seating of prosthesis. Die height above the undercut in both condensation silicones was accompanied by significant changes after disinfection, which interferes with the appropriate seating of prosthesis.

In the study of Matyas et al. which evaluated the effect of disinfectants on dimensional accuracy of impression materials, they immersed the condensation silicones of polyvinyl siloxane and irreversible hydrocolloids (alginates) in ortho phenyl phenol, glutaraldehyde, sodium hypochlorite, phenol and formaldehyde for 10 minutes. They checked the prepared casts for 3 dimensions: diameter in the gingival margin of preparation, the die height, and the diameter in occlusal area.

The results revealed that the diameter of the marginal area and the height of the die of all condensation silicones, which had been immersed in mentioned disinfectants, had increased; but 0.5% sodium hypochlorite showed less increase than other disinfectants.  

The die diameter in occlusal area showed increase in all disinfectants, excluding ortho phenyl phenol, and again the least increase was reported from sodium hypochlorite. These results are only consistent with the height of the die without undercut in Irasil group and the die height above the undercut in both materials; although none of them were statistically significant [18].

In a study, Balkaya et al. disinfected the condensation silicones with chlorine compounds for 15 minutes. The cast of the obtained impressions were at prepared at different time intervals (45 minutes, 60 minutes, and 24 hours later).

The results represented that disinfection of condensation silicones and the time intervals does not affect the dimensional stability [19]. But in the current research, it was observed in both Speedex and Irasil groups only about the diameter and height of the die without undercut. Also there was a significant difference in the height of die above the undercut in both condensation silicone groups.

The distance between the two dies was significantly different in Speedex group, while in terms of the diameter below the undercut only Irasil group showed significant difference. Regarding the fact that immersion duration was a bit different in the two studies, this difference can be contributed to the different disinfection time spans.

In a study performed by Martin et al. on the effect of disinfection on the stability of the impression materials, they used two condensation silicones of Optosil-Xantopren and Aquasil and measured the dimensions at three different intervals (immediately after impressioning, after immersion, and 24h after the impression). The results of all materials revealed that all the impressions contracted after immersion in 5.52% sodium hypochlorite; the only exception was Optosil which expanded. None of these changes were statistically significant [3].

According to the unequal laboratory conditions (such as humidity and temperature), as well as the lack of the impact of factors involved in oral impression (like blood and saliva), a similar study is suggested to be conducted in a completely controlled laboratory conditions and in oral environment, as well.

In this study, the die dimensions were measured and the casts were investigated. Therefore, the impact of other factors (such as preparation of wax patterns, cylindering stages, type of restorations’ metal alloy) featuring with seating of restoration was not studied. 

## Conclusion

Significant dimensional changes about Speedex including decrease in distance between the two dies and increasing die height above the undercut may prevent the proper seating of the prosthesis especially in cases of fixed partial prosthesis (bridge). About Irasil, increasing die height above the undercut and decreasing die width below the undercut happened. So its negative effect should be considered during impression of the teeth prepared with undercut that might result in serious distortions in impressions. The significant dimensional changes after disinfection can be contributed to the chemical nature of the disinfectant and its reaction with the impression material.

## References

[1] Miller CH (1998). Infection control & management of hazardous material for the dental team.

[2] Merchant VA, Molinari JA (1989). Infection control in prosthodontics: a choice no longer. Gen Dent.

[3] Martin N, Martin MV, Jedynakiewicz NM (2007). The dimensional stability of dental impression materials following immersion in disinfecting solutions. Dent Mater.

[4] Kalantari MH, Safari A (2009). Comparison of dimensional stability of two condensation silicone impression material in putty-wash technique. J Dent Shiraz Univ Med Sci.

[5] Melilli D, Rallo A, Cassaro A, Pizzo G (2008). The effect of immersion disinfection procedures on dimensional stability of two elastomeric impression materials. J Oral Sci.

[6] Amin WM, Al Ali MH, Al Tarawneh, Taha ST, Saleh MW, Ereifij N (2009). The effects of disinfectants on dimensional accuracy and surface quality of impression materials and gypsum casts. J Clin Med Res.

[7] Palenik CJ, Miller CH (1985). Treating highly infectious patients in the dental office. J Indiana Dent Assoc.

[8] Taylor RL, Wright PS, Maryan C (2002). Disinfection procedures: their effect on the dimensional accuracy and surface quality of irreversible hydrocolloid impression materials and gypsum casts. Dent Mater.

[9] Adabo GL, Zanarotti E, Fonseca RG, Cruz CA (1999). Effect of disinfectant agents on dimensional stability of elastomeric impression materials. J Prosthet Dent.

[10] Blair FM, Wassell RW (1996). A survey of the methods of disinfection of dental impressions used in dental hospitals in the United Kingdom. Br Dent J.

[11] Hutchings ML, Vandewalle KS, Schwartz RS, Charlton DG (1996). Immersion disinfection of irreversible hydrocolloid impressions in pH-adjusted sodium hypochlorite. Part 2: Effect on gypsum casts. Int J Prosthodont.

[12] Wadhwani CP, Johnson GH, Lepe X, Raigrodski AJ (2005). Accuracy of newly formulated fast-setting elastomeric impression materials. J Prosthet Dent.

[13] Abdelaziz KM, Hassan AM, Hodges JS (2004). Reproducibility of sterilized rubber impressions. Braz Dent J.

[14] Lepe X, Johnson GH, Berg JC, Aw TC, Stroh GS (2002). Wettability, imbibition, and mass change of disinfected low-viscosity impression materials. J Prosthet Dent.

[15] Shillingburg Jr HT, Sather DA, Wilson EL, Cain JR, Mitchell DL, Blanco LJ, Kessler JC (2012). Fundamentals of fixed prosthodontics.

[16] James C (2010). Practical infection control in dentistry.

[17] Sadr J, Naserkhaki M (2009). Evaluation of effect of sodium hypochlorite disinfectant solution dimensional stability of putty- wash silicone impression material. Thesis No. 3462.

[18] Matyas J, Dao N, Caputo AA, Lucatorto FM (1990). Effects of disinfectants on dimensional accuracy of impression materials. J Prosthet Dent.

[19] Balkaya MC, Akgungor G, Pamuk S, Gur H, Kutay O 0434 Effect of disinfection on dimensional stability of C-silicone impression material. iadr.confex.com/iadr/2004Hawaii/techprogram/abstract_46319.htm.

